# An Integrated Proteomics and Metabolomics Strategy for the Mechanism of Calcium Oxalate Crystal-Induced Kidney Injury

**DOI:** 10.3389/fmed.2022.805356

**Published:** 2022-03-03

**Authors:** Songyan Gao, Yufan Chao, Na Li, Henghui Li, Hongxia Zhao, Xinru Liu, Wei Chen, Xin Dong

**Affiliations:** ^1^Institute of Translational Medicine, Shanghai University, Shanghai, China; ^2^School of Medicine, Shanghai University, Shanghai, China; ^3^School of Life Sciences, Shanghai University, Shanghai, China; ^4^Department of Nephrology, Changhai Hospital, Naval Military Medical University, Shanghai, China

**Keywords:** proteomics, metabolomics, calcium oxalate crystal, kidney injury, mechanism

## Abstract

Renal fibrosis is the pathological repair reaction of the kidney to chronic injury, which is an important process of chronic kidney disease (CKD) progressing to end-stage renal failure. Nephrolithiasis is one of the most common renal diseases, with waist and abdomen pain, hematuria, urinary tract infection, and other clinical symptoms, which can increase the risk of renal fibrosis. Oxalate crystal-induced kidney injury is an early stage of nephrolithiasis; it is of great significance to explore the mechanism for the prevention and treatment of nephrolithiasis. A rodent model of calcium oxalate (CaOx) crystal-induced kidney injury was used in the present study, and a network analysis method combining proteomics and metabolomics was conducted to reveal the mechanism of crystal kidney injury and to provide potential targets for the intervention of nephrolithiasis. Using the metabolomics method based on the UHPLC-Q/TOF-MS platform and the iTRAQ quantitative proteomics method, we screened a total of 244 metabolites and 886 proteins from the kidney tissues that had significant changes in the Crystal group compared with that in the Control group. Then, the ingenuity pathway analysis (IPA) was applied to construct a protein-to-metabolic regulatory network by correlating and integrating differential metabolites and proteins. The results showed that CaOx crystals could induce inflammatory reactions and oxidative stress through Akt, ERK1/2, and P38 MAPK pathways and affect amino acid metabolism and fatty acid β-oxidation to result in kidney injury, thus providing an important direction for the early prevention and treatment of nephrolithiasis.

## Introduction

Nephrolithiasis is one of the most common diseases in urology, with waist and abdomen pain, hematuria, urinary tract infection, and other clinical symptoms, and it increases the risk of chronic kidney disease (CKD), renal fibrosis, coronary heart disease, and stroke (1–3). Renal fibrosis is the pathological repair reaction of the kidney to chronic injury, which is an important process of CKD progressing to end-stage renal failure (4). Nephrolithiasis and renal fibrosis tend to develop over many years, making it difficult to identify disease progression before the kidney function is severely impaired or even the failure occurs. Moreover, at the present stage, no matter nephrolithiasis or renal fibrosis, its pathogenesis has not been clearly clarified. Therefore, it is urgent to strengthen research on the mechanism of renal calculus and renal fibrosis.

Recently, with rapid development in high-throughput technologies, proteomics and metabolomics have been widely used to study the mechanisms of kidney diseases (5–7), such as CKD, acute kidney injury, and diabetic kidney disease. Proteomics focuses primarily on proteins, while metabolomics focuses on downstream small-molecule metabolites. Proteins and metabolites are the important intermediate molecules that connect the genotype and phenotype. A combined network analysis of proteomics and metabolomics is an effective tool for exploring the potential mechanism of gene phenotype (8). Wettersten et al. have extensively analyzed human renal cell carcinoma tissues using proteomics and metabolomics techniques and found that the glutathione pathway is upregulated and the β-oxidation pathway is inhibited in renal cell carcinoma cells, which provides a solid theoretical basis for the development of novel antimetabolic therapeutic strategies for renal carcinoma (9).

Nephrolithiasis affects the life quality of patients and aggravates the economic burden of people because of its high prevalence and high recurrence rate. At present, research on nephrolithiasis disease mainly focuses on the mechanism of stone formation (10, 11), early diagnosis technology (12, 13), treatment strategy (14, 15), etc. There is still no unified conclusion on the pathogenesis of kidney stones due to the complexity of their formation mechanism. In this study, we focused on an integrated analysis strategy of proteomics and metabolomics, rather than single proteomics (16) or metabolomics (17), to analyze the internal changes of mice with kidney stones in many aspects, so as to overcome the limitations of single omics and aim to further reveal the changes of metabolic regulatory networks in mice with kidney stones.

## Materials and Methods

### Chemicals and Reagents

Glyoxylic acid (50% in water) was purchased from TCI (Tokyo, Japan). The iTRAQ kit was purchased from AB SCIEX (113 Foster City, CA, USA). Methanol and acetonitrile were purchased from Merck (Darmstadt, Germany). Ultrapure water was prepared using a Milli-Q water purification system (Millipore Corp., Billerica, MA, USA). Formic acid, ammonium formate, and 2-chloro-L-phenylalanine were obtained from Sigma-Aldrich (St. Louis, MO, USA).

### Animal Experiment and Sample Collection

All animal experiments were performed in accordance with the National Institutes of Health (NIH) Guide for the Care and Use of Laboratory Animals. About 40 wild-type male C57BL/6 mice (7–8 weeks) were purchased from Shanghai SLAC Laboratory Animal Co., Ltd. (Shanghai, China). After conditional housing for 1 week, these mice were randomly divided into the Control group and Crystal group, with 20 mice in each group. All mice had free access to food and water. The mice in the Crystal group were i.p. injected with glyoxylate at a dosage of 120 mg/kg once daily for 5 days, and the mice in the Control group were i.p. injected with the same volume of saline. At the end of the animal experiment, the blood was drawn from the eyeball of all mice, and then all kidneys were harvested after *in situ* cardio perfusion. The three kidney tissues from each group were fixed in 4% paraformaldehyde for further histological analysis; others were immediately stored at −80°C. After keeping the same at 4°C for 2 h, the blood was centrifuged at 3,500 rpm for 15 min, and then the serum was collected at −80°C.

### Histological and Biochemistry Analysis

The fixed tissues were processed for paraffin embedding, and 3–4 μm sections were prepared and stained with Von Kossa. Renal calcium deposition at the junction of the cortex and medulla was observed using a microscope. The serum creatinine and urea nitrogen content were determined using the Mindray biochemical analyzer BS-380 (Guangdong, China). Calcium content in the fresh kidney tissue was measured using the Calcium Assay kit by the colorimetric method, and the activities of glutathione peroxidase (GSH-Px), superoxide dismutase (Sod), and malondialdehyde (MDA) were determined using the Commercial kit from Nanjing Jiancheng (Jiangsu, China). The levels of Interleukin-6 (Il-6), Interleukin-10 (Il-10), Interleukin-1β (Il-1β), tumor necrosis factor-α (Tnf-α), intercellular cell adhesion molecule (Icam), and vascular cell adhesion molecule (Vcam) were determined using ELISA kits purchased from Nanjing Jiancheng (Jiangsu, China). Immunohistochemical staining for target proteins was performed on kidney sections using the DAB Detection kit from Beyotime Biotechnology (Jiangsu, China). All of the primary antibodies come from the Proteintech Group (Rosemont, IL, USA).

### Metabolomics Analysis

Eight kidney tissues from each group were used for metabolomics analysis. The frozen tissues were weighed and then homogenized for 2 min with 1.5 ml 80% methanol solution containing 4 μg/ml 2-chloro-L-phenylalanine as the internal standard. Then, the homogenate was centrifuged at 13,000 rpm for 15 min at 4°C. The supernatant was transferred to injection vials and was mixed with 10-μl aliquot supernatant as the quality control (QC) sample.

Metabolomics analysis was performed on the Agilent 1290 Infinity LC system equipped with an Agilent 6538 Accurate Mass Quadrupole Time-of-Flight mass spectrometer (Agilent, USA) (UHPLC-Q/TOF-MS) using a Waters XBridge BEH Amide column (2.5 μm, 100 × 2.1 mm, Waters, Milford, MA, USA) and a Waters XBridge BEH C18 Column (2.5 μm, 100 × 2.1 mm, Waters, Milford, MA, USA). The mobile phase of an amide column consisted of a water solution containing 0.1% formic acid and 10 mM ammonium formate (phase A) and an acetonitrile solution containing 0.1% formic acid (phase B). The elution conditions were 0–1 min, 95%B; 1–3 min, 95–85%B; 3–13 min, 85–60%B; and the post time was 5 min for equilibrating the system. The flow rate was set at 0.4 ml/min; the column temperature was set at 25°C; and the injection volume was 4 μl. The mobile phases of the C18 column were 0.1% formic acid (phase A) and acetonitrile modified with 0.1% formic acid (phase B). The elution conditions were 0–2 min, 2%B; 2–10 min, 2–66%B; 10–17 min, 66–98%B; 17–19 min, 98%B; and the post time was 5 min. The flow rate was set at 0.4 ml/min; the column temperature was set at 25°C; and the injection volume was 2 μl. The mass data were acquired in both ESI^+^ and ESI^−^ modes with the mass range from 50 to 1,100 Da. The capillary voltage was 4 kV in the positive mode and 3.5 kV in the negative mode, with a Gas Temp of 350°C and a Fragmentor Voltage of 120 V. The collision energy of the MS/MS ranged from 10 to 40 eV.

The tissue samples were analyzed using the abovementioned UHPLC-Q/TOF-MS method, and the QC sample was evenly inserted into the sequence to evaluate the stability of the system.

The pretreatment of mass data was based on our previously reported method (18). The open-source R software package XCMS was used for peak extraction, alignment, and integration. Then, these variables were analyzed with the SIMCA-P software (version 11.0, Umetrics, Umea, Sweden) for multivariate statistical analysis to evaluate the stability of the UHPLC-Q/TOF-MS system. The metabolic annotation was based on matching the accurate *m*/*z* values obtained from the metabolomics analysis and MS/MS spectrogram on the pooled QC sample with those of entries in the Metlin database (https://metlin.scripps.edu/). The metabolites identified based on the MS/MS spectrogram were accurately annotated, and those identified only based on *m*/*z* values (MS) were putatively annotated. Following the metabolic annotation, all the identified peaks were checked and quantified using Agilent MassHunter Profinder B.06.00. Some metabolites were reported in more than one liquid chromatography-mass spectrometry (LC-MS) method in which the good peak shape and the least coefficient of variance for the QC samples were selected.

### iTRAQ Quantitative Proteomics Analysis

Nine frozen kidney tissues from different mice in each group were used for the iTRAQ quantitative proteomic analysis based on our previous protocol (19). Randomly, three tissues from each group were pooled together as a new sample. The protein from the pooled samples was extracted, purified, and then quantified using the BCA Protein quantitative kit. About 100 μg of the protein from each pooled sample was reduced and alkylated using the Reducing Reagent and the Cysteine-Blocking Reagent in the iTRAQ buffer kit, and hydrolyzed into peptides using sequencing grade trypsin [1: 50 (w:w)]. Then, the tryptic peptides were labeled with isobaric tags in the iTRAQ Reagents kit according to the manufacturer's manual, the samples in the Control group were labeled by 113,115,117 tags and those in the Crystal group were labeled by 114,116,118 tags. The labeled peptides were mixed together and then fractioned by high pH reverse-phase liquid chromatography (pH = 10) using Agilent ZORBAX 300Extend-C18 column (5 μm, 4.6 × 250 mm). The elutions were merged into 10 fractions, which were analyzed on the Eksigent nano LC-Ultra^TM^ system tandem TripleTOF 5600^+^ (AB SCIEX, CA, USA). About 3 μl of the sample was loaded into a nano LC trap column (ChromXP C18CL, 3 μm, 350 μm × 0.5 mm) using solvent A (2% ACN with 0.1% formic acid) at a rate of 3 μl/min for 15 min and separated by a nano analytical column (ChromXP C18CL, 3 μm, 75 μm × 150 mm) under 30°C using solvent A (2% ACN and 0.1% formic acid solution) and solvent B (ACN solution with 2% water and 0.1% formic acid) at a rate of 300 nl/min with a 90 min gradient elution of 0–0.1 min, 5–10% B; 0.1–60 min, 10–28%B; 60–75 min, 28–50%B; 75–75.5 min, 50–80%B; 75.5–80 min, 80%B; 80–80.5 min, 80–5%B; and 80.5–90 min, 5%B. The mass data were collected in the ESI^+^ mode with the MS range of 350–1,250 Da and the MS/MS range of 100–1,500 Da. A maximum of 40 precursors per cycle were chosen for fragmentation using rolling collision energy with the dynamic exclusion for 18 s, and the fragments' charges were set at +2 to +5.

The mass spectra were extracted by the MASCOT distiller software (version 2.6), and the isotopes were removed. Then, all MS/MS spectra were searched in the protein database downloading from Uniprot based on Mascot (Matrix Science, London, UK; version 2.6.0). Scaffold (version Scaffold_4.8.2, Proteome Software, Inc., Portland, OR, USA) was used to identify peptides and proteins. Peptides with a Scaffold Local FDR of <1% were considered credible and proteins that had two unique peptides were considered credible. Scaffold Q+ (version Scaffold_4.8.2, Proteome Software, Inc., Portland, OR, USA) was used for the quantification of peptides and proteins. Mann–Whitney test and Benjamini–Hochberg adjustment were used to analyze the difference between the two groups.

### Integrated Ingenuity Pathway Analysis of Metabolites and Proteins

The quantified differential proteins and metabolites were analyzed using the ingenuity pathway analysis (IPA) software (Qiagen, Redwood City, CA, USA). The dataset containing ID, fold change values, and *p*-values of differentially expressed proteins and metabolites was uploaded to IPA for core analysis, in which the canonical pathways, diseases and functions, upstream regulator analysis, toxicity analysis, and network analysis associated with calcium oxalate (CaOx) crystal-induced kidney injury could be carried out. The canonical pathways display the most significant related canonical pathways (metabolic pathway and signal pathway) across the entire dataset. The analysis of diseases and functions examines the molecules in the dataset that are known to affect diseases and functions, compares the molecules' direction of change to expectations derived from the literature, and then issues a prediction for each disease and function based on the direction of change. An IPA upstream regulator analysis is used to identify upstream regulators and predict whether they are activated or inhibited based on expected causal effects between upstream regulators and targets, given the observed molecular changes in the dataset. Tox Lists are lists of molecules that are known to be involved in a particular type of toxicity. The IPA-Tox analysis uses Toxicity Functions in combination with Toxicity Lists to link experimental data to clinical pathology endpoints, understand a pharmacological response, and support the mechanism of action and mechanism of toxicity hypothesis generation. The network analysis module analyzes the interaction between molecules with common functions to build networks. The whole core analysis relies on the Ingenuity® Knowledge Base, which is hand curated by IPA content scientists according to the literature. The IPA scores are mainly based on the value of *p* and *z*-score, and the values of *p* are calculated based on the Right-Tailed Fisher's Exact Test algorithm and reflect whether the association between a set of meaningful molecules in the experiment and known processes/pathways/transcription comes from random matching. While the *z*-score algorithm is designed to reduce the chance that random data will generate significant predictions. If the *z* score of molecule/function is greater than 2, it is generally considered to be significantly activated, and if it is less than −2, it is considered to be significantly inhibited.

## Results

### Histological and Biochemical Analyses

**Figure 1:**
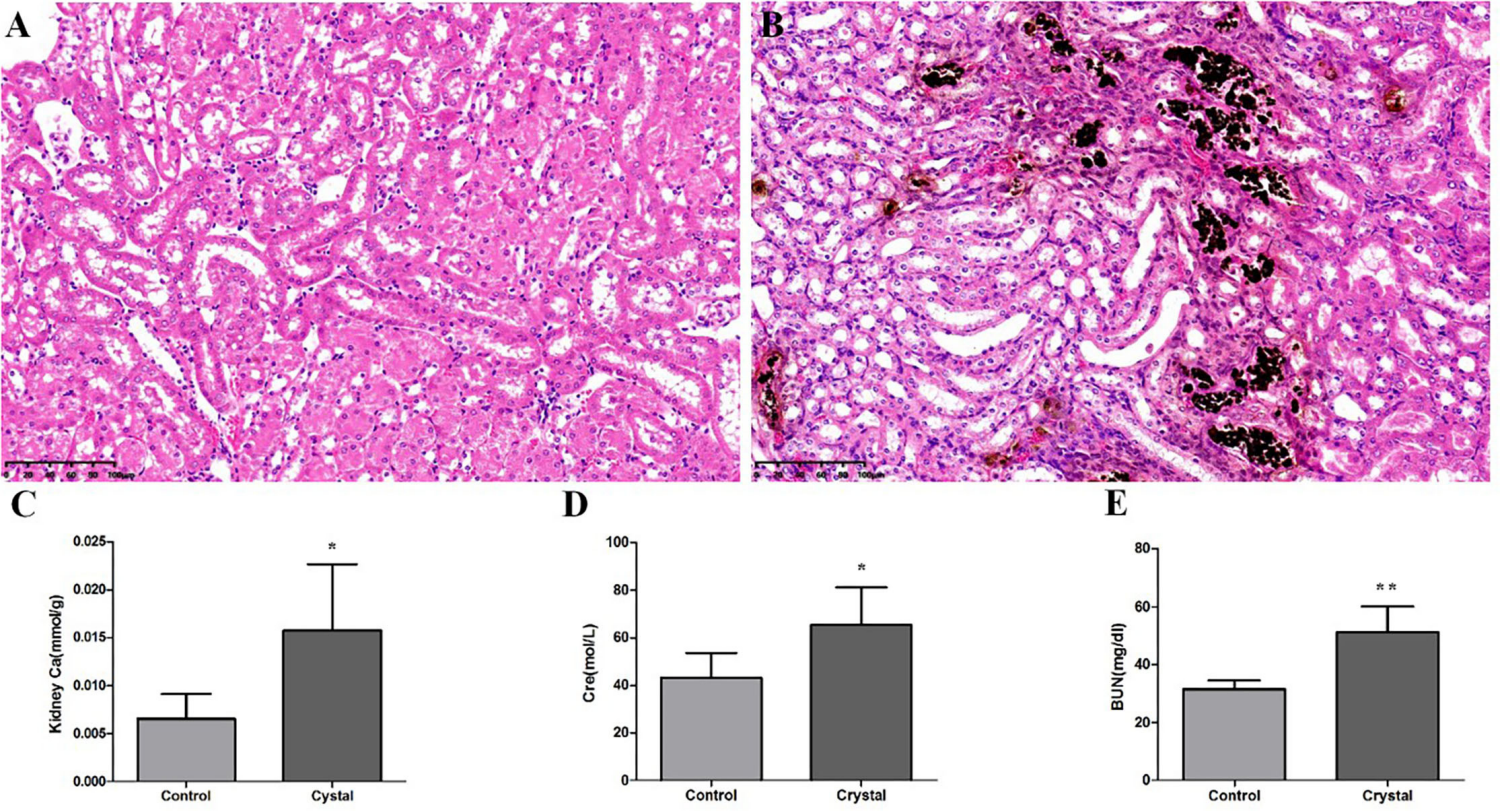
The Von Kossa Staining sections (×400) (*n* = 3) of kidney calcium and biochemical analyses (*n* = 5) in the Control group and Crystal group. **(A)** Kidney slice stained with Von Kossa of the Control group, **(B)** Kidney slice stained with Von Kossa of the Crystal group, **(C)** Kidney calcium content of the Control group and Crystal group, and **(D,E)** the serum creatinine and blood nitrogen levels of the Control group and Crystal group. Data are expressed as mean ± SD, *p < 0.05 compared with the Control group, **p < 0.01 compared with the Control group.

Von Kossa staining of kidney tissues at the junction of the cortex and medulla showed obvious calcium deposition in the Crystal group (Figures 1A,B), and the renal calcium content in the Crystal group was significantly higher than that in the Control group (Figure 1C). Meanwhile, elevated serum creatinine and blood urea nitrogen levels are measured after glyoxylate injection (Figures 1D,E). Based on the immunohistochemical staining, a significantly higher expression of different proteins, including Serpina3, Anxa3, Cd44, galectin-1 (Lgals1), Krt8, Lcn2, Cd14, and S100a4, was detectable, after oxalate crystal-induced renal injury (Supplementary Figure 1).

### Metabolic Profiling Analysis

**Figure 2:**
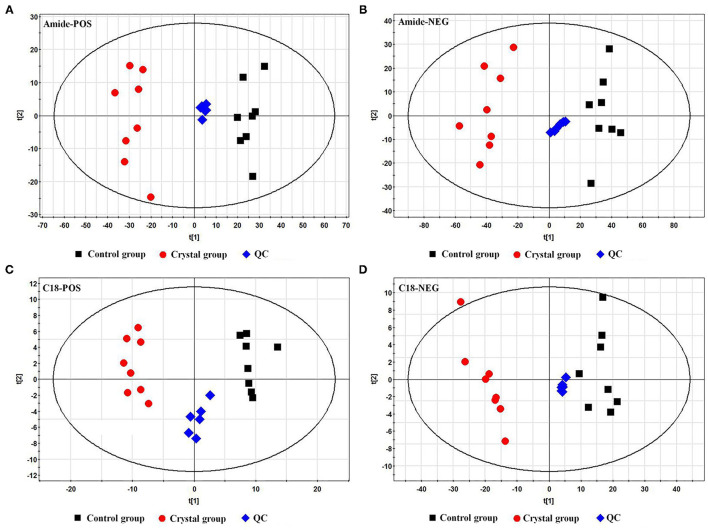
Principal component analysis (PCA) score plots of the Control group, Crystal group, and QC samples based on metabolites. **(A)** A plot in the ESI^+^ mode using an amide column, **(B)** a plot in the ESI^−^ mode using an amide column, **(C)** a plot in the ESI^+^ mode using a C18 column, and **(D)** a plot in the ESI^−^ mode using a C18 column.

The representative total ion chromatograms (TICs) of tissue samples and QC samples acquired using the abovementioned UHPLC-Q/TOF-MS methods are shown in Supplementary Figure 2. About 2,219 (Amide pos), 690 (Amide neg), 1,559 (C18 pos), and 416 (C18 neg) features were extracted using the XCMS package. The features were imported to SIMCA-P for a multivariate statistical analysis after normalization, then the unsupervised principal component analysis (PCA) was used to observe the aggregation of QC samples and the separation trend between the Control group and Crystal group. As shown in PCA score plots (Figure 2), the QC samples gathered well and the Crystal group was obviously separated from the Control group. These results indicated that the system is stable and the metabolic profile of the Crystal group is significantly different from that of the Control group. After data preprocessing according to the abovementioned method, 368 metabolites were identified and quantified. Compared to the Control group, there were 244 metabolites, which were changed more than 1.3-folds and the FDR adjusted *p*-values were <0.05, 119 metabolites were upregulated and 125 ones were downregulated in the Crystal group. Different metabolites and proteins are shown in Supplementary Materials.

### Proteomic Analysis

In this iTRAQ experiment, we totally identified 187,510 spectra and 5,191 proteins. There were 886 proteins, which showed a significant change between the Crystal group and Control group (fold change > 1.3, adjusted *p* < 0.05). Among these, 697 proteins were upregulated and 189 ones were downregulated compared with the Control group. Different proteins and metabolites are shown in Supplementary Materials .

### Integrated IPA of Metabolites and Proteins

**Figure 3:**
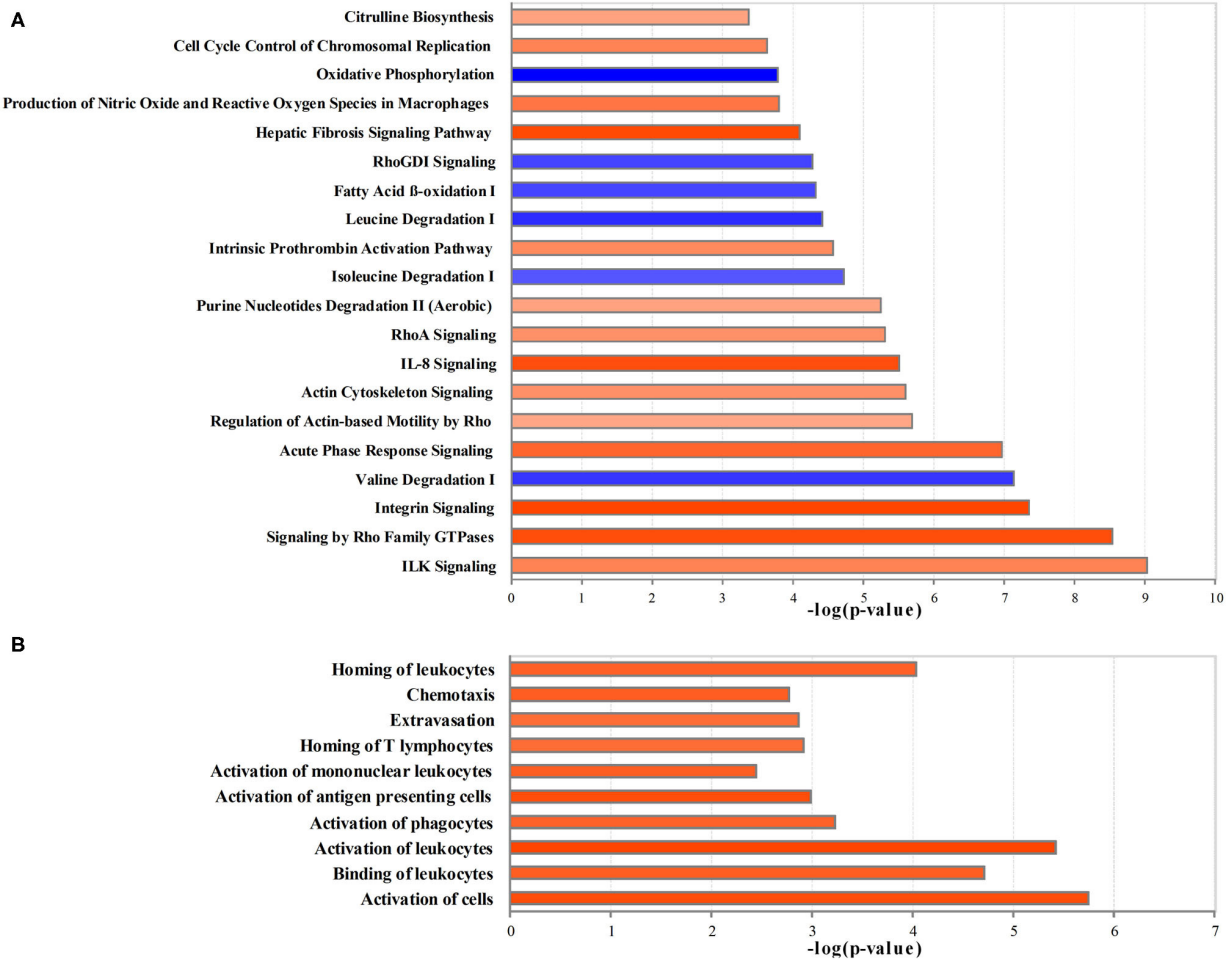
The creditable canonical pathways and disease or functional annotation based on the differentially expressed proteins and metabolites (|*z*-score>2| and *p* < 0.05). **(A)** Canonical pathways. **(B)** Disease or functional annotation. Orange represents activation (*z*-score >2); blue represents inhibition (*z*-score < −2), and the darker color represents a more significant activation or inhibition.

The ingenuity pathway analysis was used to integrate 886 differential proteins and 244 differential metabolites for core analysis. Figure 3A shows the top most 20 significant dysregulated canonical pathways (|*z*-score > 2|). Integrin-linked kinase (ILK) signaling, signaling by Rho family GTPases, integrin signaling, acute phase response signaling, the regulation of actin-based motility using rho, actin cytoskeleton signaling, IL-8 signaling, RhoA signaling, purine nucleotide degradation II (aerobic), intrinsic prothrombin activation pathway, hepatic fibrosis signaling pathway, the production of nitric oxide (NO) and reactive oxygen species (ROS) in macrophages, cell cycle control of chromosomal replication, citrulline biosynthesis were significantly activated, and valine degradation, isoleucine degradation, leucine degradation, fatty acid β-oxidation (FAO), RhoGDI signaling, and oxidative phosphorylation were significantly inhibited.

In comparison with the Control group, the proteins and metabolites with the expression differed by 2- or 0.5-fold in the Crystal group were selected for the analysis of diseases and functions, toxicity analysis, upstream analysis, and network analysis. Per disease and function analysis, the following process was significantly increased in the Crystal group: the activation of cells, binding of leukocytes, activation of leukocytes, activation of phagocytes, activation of antigen presenting cells, activation of mononuclear leukocytes, homing of T-lymphocytes, extravasation, chemotaxis, and homing of leukocytes, which were categorized as cell-to-cell signaling and interaction, immune cell trafficking, and inflammatory response (Figure 3B).

An upstream regulator analysis could predict the upstream regulators that can regulate the changes for the submitted molecules. Il6, Tnf, Osm, Il10, Il1B, Csf2, Nos2, Cebpa, and Agt were determined to be upstream cytokines, an enzyme, a transcription regulator, or a growth factor. The target molecules in the dataset are shown in Table 1.

**Table 1:** Upstream regulators for proteins and metabolites, which showed over 2-folds between the Crystal group and Control group (*z*-score>2).

**Upstream regulator**	**Expr fold change**	**Molecule type**	**Predicted activation state**	**Activation z-score**	**^a^Flags**	**^b^p-value of overlap**	**Target molecules in data set**
Il6		Cytokine	Activated	2.598	Bias	0.000000943	5-hydroxytryptamine, Akr1b10, Arg1, Cd14, Cd44, Chil3/Chil4, Hla-Dqa1, Hp, Krt8, Lcn2, Lgals1, Lrg1, Serpina3
Nos2		Enzyme	Activated	2.433		0.0000408	Cd14,Cd44,Chil3/Chil4,citrulline,Lcn2,Serpina3
Cebpa		Transcription regulator	Activated	2.407		0.000494	Akr1b10,Arg1,Cd14,Hp,Lcn2,Lgals1,Mup1 (includes others)
Osm		Cytokine	Activated	2.578		0.00161	Akr1b10,Anxa3,Arg1,Hp,Lcn2,Lrg1,Serpina3
Tnf		Cytokine	Activated	3.368		0.00637	5-hydroxytryptamine,Akr1b10,Arg1,Cd14,Cd44, citric acid, Fth1, Hp, Krt8, Lcn2, Lrg1, phosphorylcholine, Serpina3
Agt	1.39	Growth factor	Activated	2.63	Bias	0.00766	Anxa3, Arg1, Cd14, Cd44, Hp, Lcn2, Loxl2, Serpina3
Srebf1		Transcription regulator	Activated	2	Bias	0.00926	Alpha-hydroxyglutarate, Cd14, Chil3/Chil4, Serpina3
Cebpb		Transcription regulator	Activated	2.149	Bias	0.0108	Akr1b10, Aldh1a2, Arg1, Cd14, Hp, Lcn2
Il10		Cytokine	Activated	2.218		0.0149	Arg1,Cd14,Cd44,Chil3/Chil4,Lgals1
Il1B		Cytokine	Activated	2.264		0.0181	5-hydroxytryptamine, Arg1, Cd14, Cd44, Hp, Lcn2, Lcp1, Serpina3
Csf2		Cytokine	Activated	2.168	Bias	0.0254	Arg1,Cd14,Chil3/Chil4,Lcp1,Rbm3

a *The Bias Term is the product of the data set bias and the upstream regulator bias. When the absolute value of this term is 0.25 or higher, then the upstream regulator's prediction is considered to be biased (20). Such rows are labeled with the word “bias” in the Notes column*.

b *p-value of overlap is defined to measure the enrichment of network-regulated genes in the data set without taking into account the regulation direction. The calculation is based on the one-sided Fisher's exact test, which assumes a random data set with a constant number of genes as the null model (20)*.

The toxicity analysis showed that Anxa3, Cd14, Cd44, Lcn2, Umod, Dpysl3, hepatitis A virus cellular receptor 1 (Havcr1), Hp, Serpina3, and Lgals1 were responsible for acute renal failure (AKI), persistent renal ischemia-reperfusion injury, positive acute phase response proteins and increasing the transmembrane potential of mitochondria and mitochondrial membrane (Table 2).

**Table 2:** Toxicity analysis of proteins and metabolites, which showed over 2-fold differences between the Crystal group and Control group.

**Ingenuity toxicity lists**	**^a^-log (*p*-value)**	**^b^Ratio**	**Molecules**
Acute renal failure panel (Rat)	5.87	0.0806	Anxa3,Cd14,Cd44,Lcn2,Umod
Persistent renal ischemia-reperfusion injury (Mouse)	3.97	0.1	Dpysl3,Havcr1,Lcn2
Positive acute phase response proteins	2.42	0.0667	Hp,Serpina3
Increases transmembrane potential of mitochondria and mitochondrial membrane	1.98	0.04	Lgals1, Serpina3

a *The **–**log (p-value) reflects whether the association between differentially expressed molecules and toxicity processes comes from random matching*.

b *The ratio shows the extent of overlap of differentially expressed molecules with the total molecules in the Toxicity List*.

**Figure 4:**
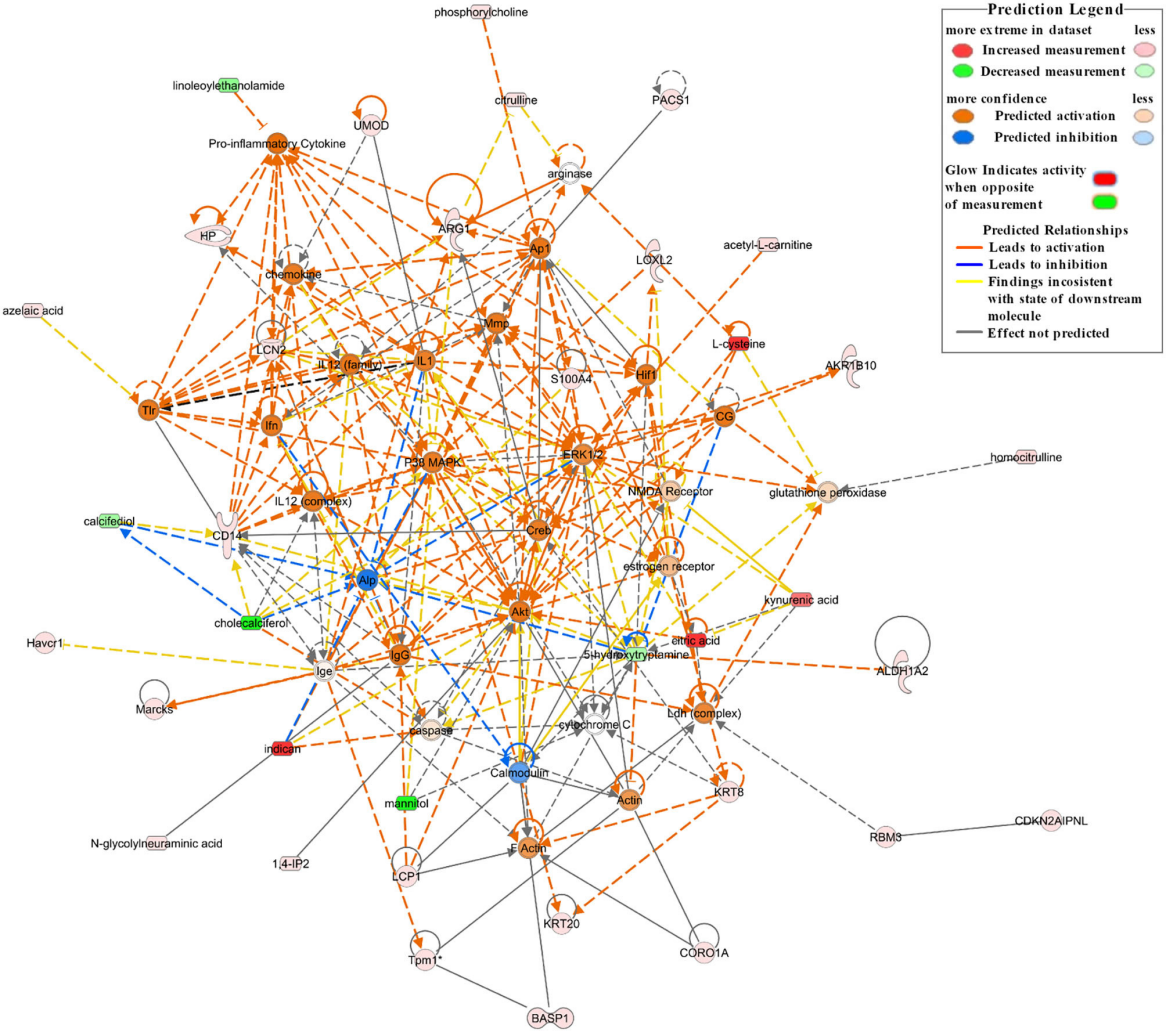
The top network associated with immunological disease, inflammatory disease, and inflammatory response based on the significantly differentially expressed proteins and metabolites between the Crystal group and Control group. Red shapes represent upregulated molecules and green shapes represent downregulated molecules. Prediction legends are annotated in the legend box.

A network analysis of significantly differentially expressed proteins and metabolites is shown in Supplementary Table 1 (score > 20). The credible networks were related to immunological disease, inflammatory disease, inflammatory response, lipid metabolism, molecular transport, small molecule biochemistry, amino acid metabolism, and gastrointestinal disease. The top 2 score networks were associated with immunological disease, inflammatory disease, and inflammatory response and were merged together. The merged top score network demonstrated that Arg1, Basp1, Cd14, Havcr1, Hp, Lcn2, L-plastin (Lcp1), Marcks, Pacs1, Tpm1, Umod, Akr1b10, Aldh1a2, Cdkn2aipnl, coronin 1A (Coro1a), Krt20, Krt8, Loxl2, Rbm3, S100a4, acetyl-L-carnitine, citric acid, L-cysteine, homocitrulline, citrulline, indican (indoxyl sulfate), kynurenic acid, phosphorylcholine, 1,4-IP2, and N-glycolylneuraminic acid in our dataset were significantly upregulated in the Crystal group. These molecules were in interaction with each other through Akt, ERK1/2, p38 MAPK, and pro-inflammatory cytokines, and the molecular activity predictor (MAP) predicted that the linkers were activated (Figure 4).

### Validation of Inflammation and Oxidative Stress-Related Molecules

**Figure 5:**
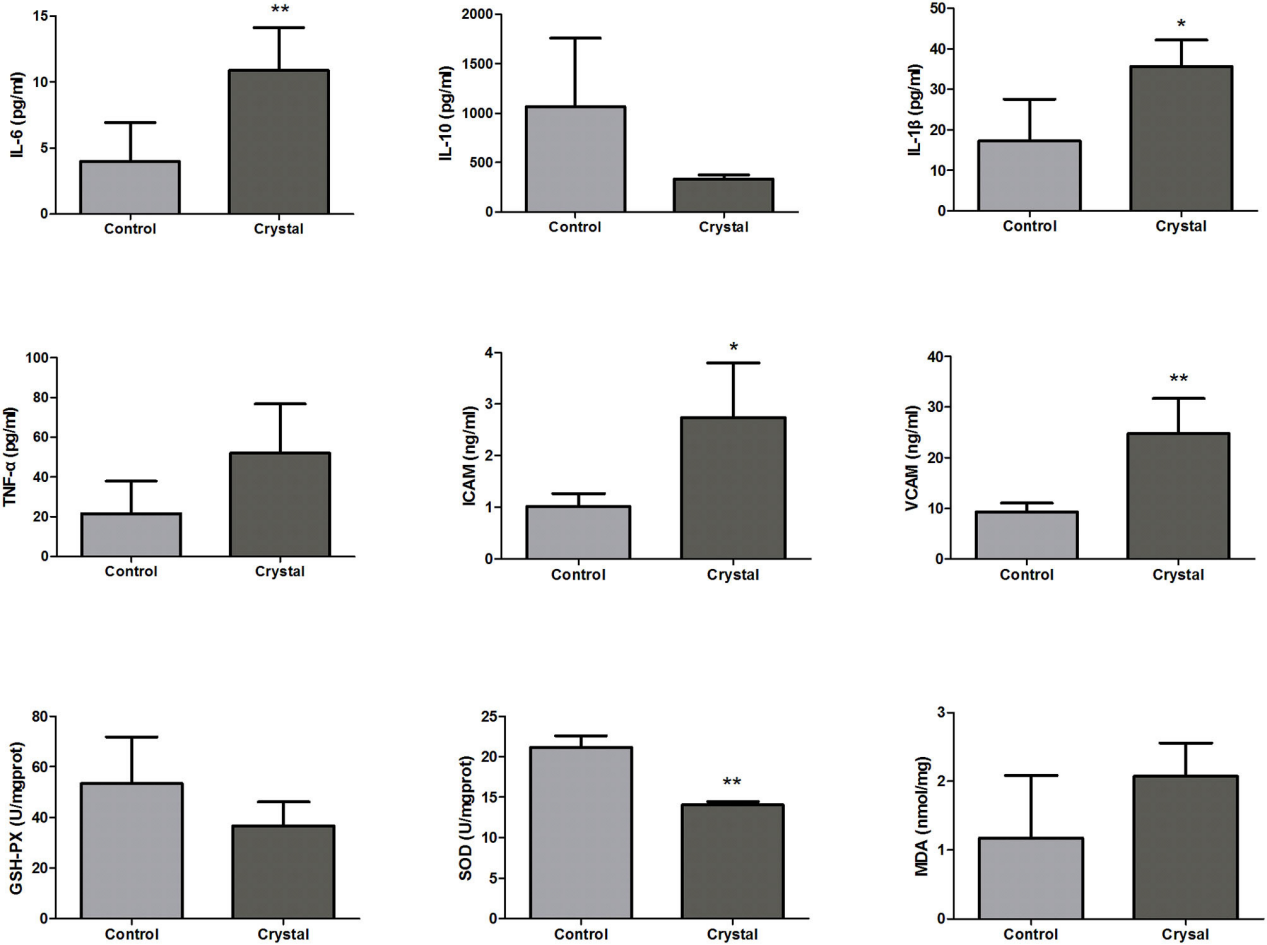
The levels or activities of Interleukin-6 (Il-6), Interleukin-10 (Il-10), Interleukin-1β (Il-1β), tumor necrosis factor-α (Tnf-α), intercellular cell adhesion molecule (Icam), vascular cell adhesion molecule (Vcam), glutathione peroxidase (GSH-Px), superoxide dismutase (Sod), and malondialdehyde (MDA) in renal tissues of the Control group and Crystal group (*n* = 5). *p < 0.05 compared with the Control group, **p < 0.01 compared with the Control group.

According to the pathway analysis, upstream regulators, and network analysis, the inflammatory cytokines, adhesion molecules, and oxidative stress indexes in renal tissues were measured to characterize the inflammation and oxidative stress state. The levels or activities of Il-6, Il-10, Il-1β, Tnf-α, Icam, Vcam, GSH-Px, Sod, and MDA in renal tissues are shown in Figure 5.

The levels of pro-inflammatory factors, Il-6, Il-1, and Tnf-α, and the adhesion molecules, Icam and Vcam, were increased in the Crystal group compared with the Control group; and the level of anti-inflammatory factor Il10 was decreased, indicating that the CaOx crystals could induce an inflammatory response. The level of MDA was increased in the Crystal group and the activities of GSH-Px and Sod were decreased, indicating that CaOx crystals induced the oxidative stress injury. Unfortunately, the differences of Il-10, Tnf-α, GSH-Px, and MDA between the two groups were not significant, which might be due to the small sample size.

## Discussion

To clarify the pathogenesis of kidney stone diseases, a combination analysis of proteomics and metabolomics of kidney tissues was conducted in this study, which was mutually verified and complementary, so as to more systematically and comprehensively analyze the function and regulatory mechanism of biomolecules and provide theoretical support for the prevention and treatment of kidney stone diseases. Most kidney stones are partially or completely composed of CaOx (21), therefore, a CaOx crystal renal injury mouse model induced by glyoxylate was conducted in our research (22).

In comparison with mice in the Control group, the proteins and metabolites of mice in the Crystal group were significantly changed. Canonical pathway analysis of the IPA software showed that multiple inflammation-related pathways (ILK signaling, integrin signaling, actin cytoskeleton signaling, Rho family GTPases and RhoA signaling, and IL-8 signaling), oxidative stress (the production of NO and ROS in macrophages, acute phase response signaling) were significantly activated; and branched chain fatty acid degradation and FAO were significantly downregulated. According to analyses of diseases and functions, cell-to-cell signaling and interaction, immune cell trafficking, and inflammatory responses were increased in the Crystal group compared with those in the Control group. Per network analysis, Cd14, Havcr1, Hp, Lcn2, Umod, Lcp1, Coro1a, Krt8, Loxl2, and S100a4 were upregulated and were in interaction with each other by activating Akt, ERK1/2, p38 MAPK, and pro-inflammatory cytokines and were related to immunological disease, inflammatory disease, and inflammatory response. Among these network nodes, Cd14, Lcn2, Umod, Havcr1, and Hp were responsible for AKI, persistent renal ischemia-reperfusion injury, and positive acute phase response proteins.

Cd14 is a pro-inflammatory protein and is involved in the innate immune system as a co-receptor for Toll-like receptors (23). A cohort study showed that a level of pro-inflammatory Cd14 was elevated in patients with renal dysfunction; this may be because of the increased inflammatory status that contributed to the development of kidney dysfunction (24). Cd44 is a ubiquitously expressed transmembrane glycoprotein and also modulates the pro-inflammatory signaling as a unique receptor complex with Toll-like Receptor 4 and MD-2 (25, 26). Havcr1, also known as T-cell immunoglobulin and mucin domain 1 (TIM-1) or kidney injury molecule (KIM-1), is a member of the TIM gene family, which is widely involved in the regulation of immune cell activity (27, 28). With various forms of injury, KIM-1 expression is markedly upregulated in the proximal tubule epithelial (29–32). KIM-1 contains an immunoglobulin domain and O-glycosylated mucin subdomains (29). The structure suggests that KIM-1 is an epithelial cell adhesion molecule and it may be involved in surface adhesion interactions of the proximal tubule epithelial cells in the postischemic kidney (30). Neutrophil gelatinase-associated lipocalin (Ngal, Lcn2), a small protein of the lipocalin family, was generally considered as a biomarker of acute renal injury (33, 34). A genome-wide analysis of the kidney showed that Lcn2 was the top upregulated gene in the papillary tissue from Randall's plaques region, which was considered to be the focus of renal stone formation (35). Ngal is expressed in neutrophils and epithelial cells and is related to inflammation and acute kidney injury (36). Ngal in the kidney may come from renal tubular cells and neutrophils infiltrating the kidney (36, 37). Uromodulin, also known as Tamm–Horsfall protein, was exclusively expressed in renal epithelial cells by Umod and is associated with the estimated glomerular filtration rate (eGFR) (38, 39). Some Umod risk variants could increase uromodulin expression and then induce salt-sensitive hypertension and kidney damage (40). The S100a4 protein, several S100 super families, could amplify the inflammatory response through binding to the specific receptors on immune cells to promote the activation of innate immunity, cell differentiation, cell death, or the secretion of inflammatory mediators as alarmin (41, 42). Anxa3 is one of the annexins, which are Ca2+ dependent phospholipid-binding proteins (43) and mainly interact with phosphatidylethanolamine. The dysregulation of Anxa3 has been reported to be associated with cancer development and progression (44). Serpina3 belongs to the family of serine protease inhibitors (45). In addition, the role of Serpina3 in the kidney may be to balance inflammation, oxidative stress, and renal fibrosis after kidney injury (46). Keratins were the intermediate filaments of the epithelial cell cytoskeleton and were expressed in an organ and epithelial cell-specific manner (47). Krt8 is a type II keratin and is expressed in all epithelial cells of the nephron (48). Krt8 expression was significantly increased in the different models of renal tubular injury and considered as a marker of epithelial cell stress injury (49, 50). Lgals1, a carbohydrate-binding protein, is significantly increased in the RCC cancer tissue and is implicated in cancer cell proliferation, invasion (51, 52). It was reported that Lgals1 can trigger an apoptotic program involving an increase of mitochondrial membrane potential and play a key role in tumor-immune escape by killing antitumor effector T-cells (53). A recent study showed that Lgals1 was continuously highly expressed in glomeruli in rat models and populations of CKD and showed a significant negative correlation with eGFR, suggesting that Lgals1 might have a key role in the injury process of the glomerulus (54).

It is noteworthy that several tryptophan metabolites, indoxyl sulfate, kynurenic acid, and xanthurenic acid showed an approach or exceeding 10-fold increases in the Crystal group. Most of the tryptophans are metabolized by the kynurenine metabolic pathway, and kynurenic acid and xanthurenic acid were the metabolites of a kynurenine metabolic pathway. Tryptophan is also metabolized by bacterial tryptophan enzymes to produce the precursor indoles, then some of them were converted into indoxyl sulfate by CYP2E1 and sulfotransferases mainly in the liver (55). Indoxyl sulfate, which is a uremic toxin, shows a significant increase in the damaged kidneys (56). Recently, many reports have suggested the intestinal metabolites of tryptophan activate the aryl hydrocarbon receptor (AHR) signaling pathway (57, 58), which can induce oxidative stress and inflammation (59) and mediate renal fibrosis (60). Hippuric acid, a gut-derived uremic toxin (61), was also significantly increased (5.881-fold) in the Crystal group. The dietary polyphenols are converted into benzoic acid through multiple phenolic reaction pathways using colon bacteria, which is then conjugated with glycine by glycine-N-acyltransferase (GLYAT) to produce hippuric acid in the liver or kidney (62). A study showed that the accumulated hippuric acid could promote renal fibrosis by inducing oxidative stress (63). There are multiple uremic toxins accumulated in the crystal group, indicating that CaOx caused a decrease in the glomerular filtration rate, and the uremic toxins will further accelerate the progression of renal dysfunction.

### Changes in Inflammation-Related Signaling Pathways

As important markers of tissue injury and inflammation, adhesion molecules play an important role in the inflammatory reaction. Compared to the mouse in the Control group, pathways related to cell adhesion (e.g., ILK signaling, integrin signaling, actin cytoskeleton signaling, and Rho family GTPases and RhoA signaling) were significantly upregulated in the mice of the Crystal group. ILK is an intracellular serine/threonine protein kinase and plays an essential role in NF-κB activation. NF-κB, an important regulator of inflammation, plays a key role in regulating inflammatory responses. ILK can be activated *via* either an interaction between an extracellular matrix (ECM) with integrins or inflammatory stimulus [e.g., lipopolysaccharides (LPS) and Tnf-α] through the PI3K pathway. Activated ILK phosphorylates the p65 subunit of NF-κB, triggering NF-κB nuclear translocation and downstream gene (inflammatory cytokines, chemokines, adhesion molecules, etc.) expression. Integrins are a family of transmembrane cell surface molecules that constitute the principal adhesion receptors for an ECM and are indispensable for the existence of multicellular organisms (64). The ability of integrins, clustered within matrix adhesion sites, regulates the signal transduction pathways that not only modulate cell adhesion and motility but also gene expression. Integrins can interact with adhesion molecules (e.g., Icam and Vcam) to induce the necessary conformational changes in the cytoplasmic tail to trigger pro-inflammatory signaling. In our study, both proteomics results and biochemistry analyses showed that Icam and Vcam in the Crystal group were significantly upregulated compared with those in the Control group. The actin cytoskeleton is the fundamental structural component of eukaryotic cells, which has a role in response to abiotic and biotic stimuli (65). The actin cytoskeleton is also central to cell function in inflammation, such as integrin-mediated cell adhesion in part depends on the reorganization of the actin cytoskeleton (66, 67). Recent studies have revealed that a balanced actin filament turnover can protect epithelial barriers and attenuate tissue injury during mucosal inflammation *in vivo* (68). In our study, as shown in Figure 4, the two actin-bundling proteins Lcp1 and Coro1a were directly related to actin and significantly upregulated in the Crystal group. Lcp1 is mainly expressed in leukocytes and regulates leukocyte adhesion and signal transduction (67). Coro1a belongs to a family of evolutionary conserved actin-binding proteins. During inflammation, Coro1a is a novel regulator of β2 integrins and regulates polymorphonuclear neutrophils (PMNs) trafficking through the induction of adhesion, adhesion strengthening, spreading, and migration (69). RhoA, which is recognized as small GTPases of the Rho family, is involved in many cellular processes, including cell structure, ROS formation, cell adhesion and migration, apoptosis, actin cytoskeletal movements, and cell differentiation (70). Rho kinase (ROCK) has ROCK1 and ROCK2 subtypes, and the RhoA/ROCK signaling pathway has been proven to be associated with the level of immune system activation and the production of pro-inflammatory factors, which have many types of downstream targets, including NF-κB, myosin light chain (MLC), MLC phosphatase (MLCP), and myosin phosphatase-targeting subunit-1 (MYPT-1). As an important downstream target of ROCK, there is a strong relationship between the degree of NF-κB activation and the severity of inflammation (71).

### Changes in the Oxidative Stress-Related Pathway

Nitric oxide and ROS were mainly produced from the mitochondrial electron transport chain, NADPH oxidase, and peroxidase (72). Under normal physiological conditions, there is a balance between the production and clearance of the NO and ROS. However, when the body produces excessive ROS, which exceeds the antioxidant defense system clearance capacity, the excessive ROS may influence the cell survival through damaging or modifying the protein, nucleic acid, lipid, and other biological macromolecules, leading to tissue injury (73). The ROS generation induces the DNA oxidative injury, resulting in tubular and endothelial cell death with the activation of the pro-inflammatory factors. In addition, inflammation may further amplify oxidative stress. The interconnected process between inflammation and oxidative stress eventually resulted in kidney injury (72). Canonical pathway analysis showed the production of NO and ROS in macrophages was significantly increased, the related proteins Akt1, Apod, Apoe, Clu, Cybb, Fnbp1, Lyz, Mapk3, Ncf2, Nfkb2, Rela, Rhob, Rhoc, Rhog, and Sirpa were upregulated in the Crystal group, and Cat (proteomics data) was downregulated in the model group. In addition, as shown in Figure 5, GSH-Px and Sod were downregulated in the Crystal group and MDA was upregulated. MDA is an end product of lipid peroxidation, and the increased MDA indicated that the body was in the lipid peroxidation status. Sod, GSH-Px, and Cat are the common antioxidant substances, all of them were decreased in the Crystal group, indicating that the ROS clearance capacity of the body was decreased.

### Changes in FAO Pathways

Canonical pathway analysis of IPA showed that FAO was significantly downregulated in the Crystal group. The related proteins medium-chain specific acyl-CoA dehydrogenase (Acadm), long-chain fatty acid—CoA ligase 1 (Acsl1), methylglutaconyl-CoA hydratase (Auh), peroxisomal bifunctional enzyme (Ehhadh), hydroxyacyl-coenzyme A dehydrogenase (Hadh), isovaleryl-CoA dehydrogenase (Ivd), and non-specific lipid-transfer protein (Scp2) were downregulated in the Crystal group compared with the Control group. These enzymes are the important enzymes involved in mitochondrial FAO, especially Acadm whose deficiency is the most common disorder of FAO and one of the most common inborn errors of metabolism (74).

The most efficient ATP-generating system in cell energy metabolism is mitochondrial FAO, which can generate 106–129 ATPs, depending on the number of carbons in the FA chain. The kidney is a highly metabolic organ and uses high levels of ATP to maintain electrolyte and acid-base homeostasis and reabsorb nutrients. Energy depletion is a critical factor in the development and progression of various kidneys. Several literature studies have also revealed that FAO in a proximal tubule is a major source of ATP generation (75). FAO serves as the preferred source of ATP in the kidney and its dysfunction results in ATP depletion and lipotoxicity to elicit tubular injury and inflammation and subsequent fibrosis progression (76). Kang et al. found that defective fatty acid oxidation in renal tubular epithelial cells has a key role in kidney fibrosis development (77).

In the process of FAO, long chain fatty acids are first activated to acyl-CoA to make it permeable to the outer mitochondrial membrane (OMM) by acyl-CoA synthetases in the cytosol. Carnitine palmitoyltransferase-1 (Cpt-1), located on the OMM, catalyzes the transesterification of the acyl-CoA to acylcarnitine. Acylcarnitine is shuttled across the inner mitochondrial membrane (IMM) through carnitine–acylcarnitine translocase. Acylcarnitine is reconverted to acyl-CoA by an IMM protein Cpt-2. Therefore, Cpt-1 or Cpt-2 is critical for normal oxidation of fatty acids; several studies have revealed that the downregulated or deficient Cpt-1 or Cpt-2 is critical to the impaired FAO in diverse kidney diseases, such as ischemic, cisplatin AKI, and diabetic nephropathy (78–80). From the metabolomics data, we found that the total long-chain acylcarnitine was increased in the Crystal group than that in the Control group, which indicated that there was a disorder in the process of Cpt-2 reconverting acylcarnitine to acyl-CoA. The β-oxidation of acyl-CoA involves dehydrogenation, hydration, redehydrogenation, and thiolysis. As the important rate limiting enzymes of the first and third steps of the β-oxidation cycle, Acadm and Hadh were reduced in the Crystal group. In addition, Acsls also can convert free long-chain fatty acids into acyl-CoAs and play crucial roles in fatty acid metabolism (81). In our study, Acsl1 was downregulated in the Crystal group. On the other hand, the fatty acid metabolite phenylacetylglycine, as an important marker of impaired mitochondrial FAO (82), was upregulated (34.454-fold) in the Crystal group. In summary, the FAO in the Crystal group mice was significantly disturbed.

## Conclusion

In our study, using the combination of an integrated proteomics and metabolomics strategy and IPA bioinformatic analysis, we screened the metabolic profiling, and protein profiling was significantly changed in CaOx crystal-induced kidney injury mice and found that the CaOx crystal could induce inflammatory reactions and oxidative stress through Akt, ERK1/2, p38 MAPK pathways and affect amino acid metabolism and fatty acid β-oxidation, resulting in renal injury.

## Data Availability Statement

The original contributions presented in the study are publicly available. This data can be found here: https://www.iprox.cn/page/DSV021.html;?url=1643726541728UCyc (Password: AQKV).

## Ethics Statement

The animal study was reviewed and approved by Ethical Committee of Naval Military Medical University.

## Author Contributions

XD, WC, and XL conceived the study, developed the proposal, and supervised the results. SG performed the animal experiment, omics experiments, data analysis, and writing the manuscript. YC was responsible for the histological and biochemistry experiment and writing of the manuscript. NL and HZ assisted SG in the collection of omics data. HL helped YC to perform the histological experiment and data analysis. All authors contributed to the article and approved the submitted version.

## Funding

This work was supported by grants from Science and Technology Commission of Shanghai Municipality (14DZ2260200, the project of Shanghai Key Laboratory of Kidney and Blood Purification and 21ZR1478700).

## Conflict of Interest

The authors declare that the research was conducted in the absence of any commercial or financial relationships that could be construed as a potential conflict of interest.

## Publisher's Note

All claims expressed in this article are solely those of the authors and do not necessarily represent those of their affiliated organizations, or those of the publisher, the editors and the reviewers. Any product that may be evaluated in this article, or claim that may be made by its manufacturer, is not guaranteed or endorsed by the publisher.
