# Supplementary material for: An Integrated Proteomics and Metabolomics Strategy for the Mechanism of Calcium Oxalate Crystal-Induced Kidney Injury
Source: Front Med (Lausanne). 2022 Mar 3;9:805356. doi: 10.3389/fmed.2022.805356 (PMC8927618; doi:10.3389/fmed.2022.805356)
Supplement: Supplementary file 2 [file Data_Sheet_1.docx]

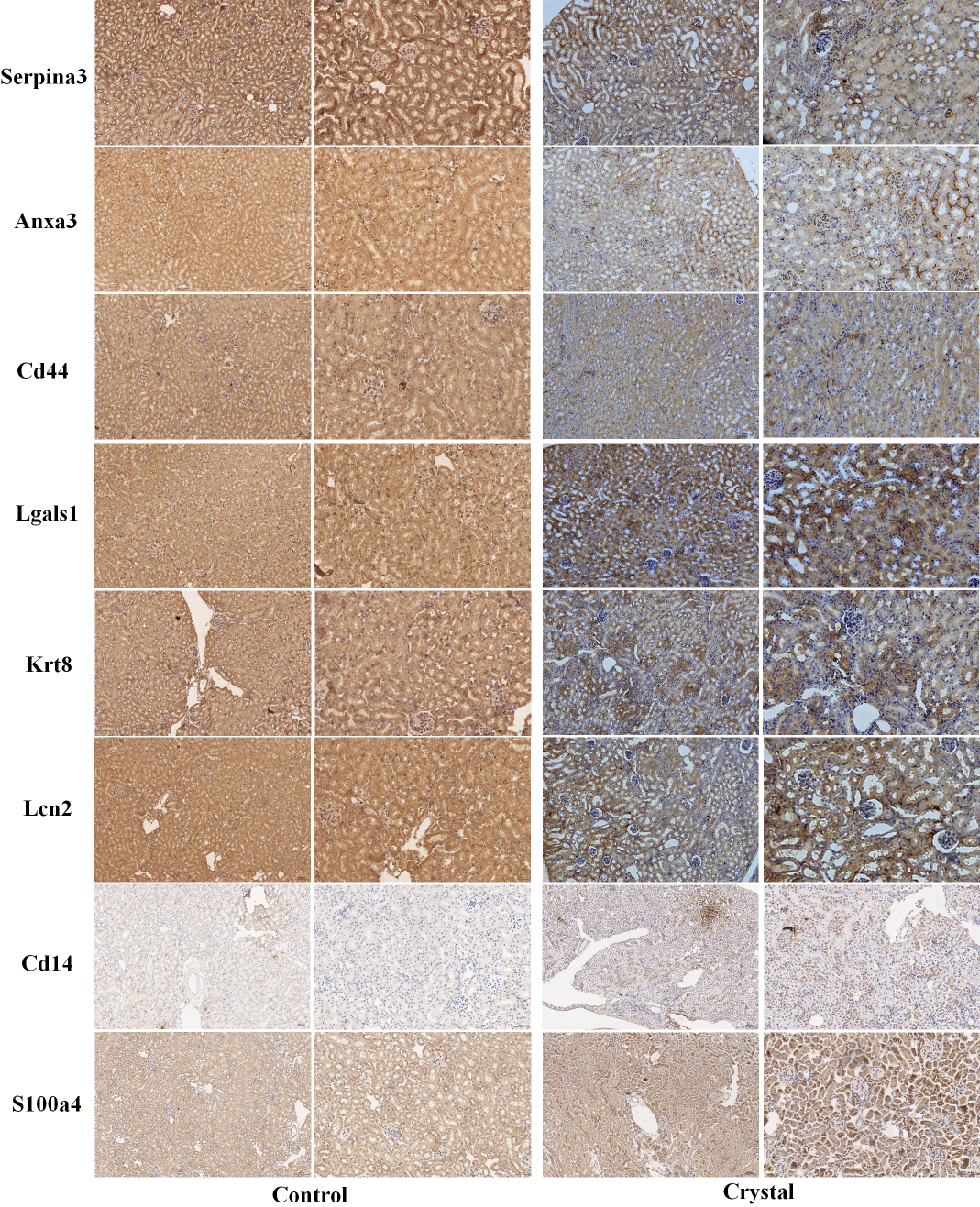


Fig.S1 The immunohistochemistry of proteins Serpina3, Anxa3, Cd44, Lgals1, Krt8, Lcn2, Cd14 and S100a4. Magnification, × 100 (left); Magnification, × 400(right)


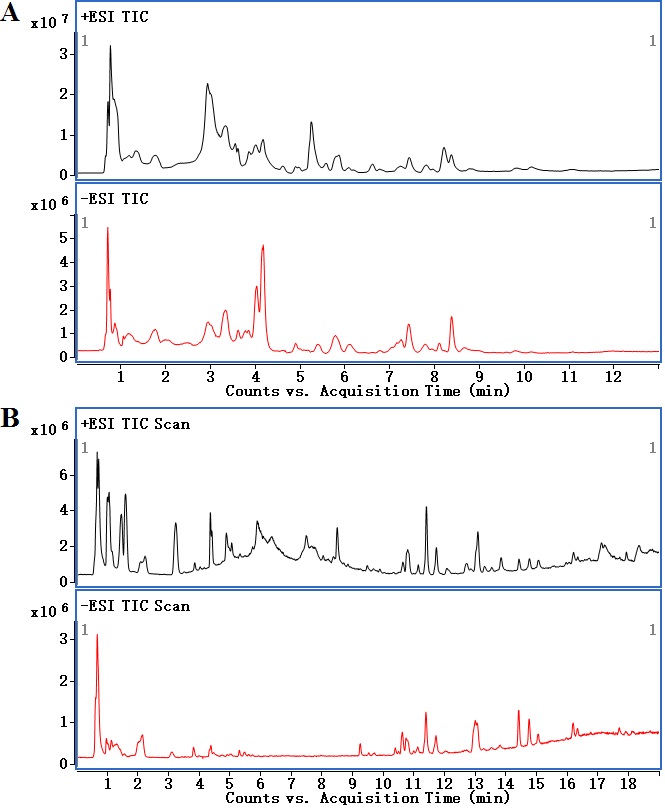


Fig.S2 Representative total ion chromatograms (TICs) of tissue samples. (A)TICs using Amide column, (B) TICs using C18 column.

Table.S1 Network analysis of proteins and metabolites whose expression differed by 2 fold or 0.5 fold in the Crystal group compared with Control group (Score ＞20). In the column of “Molecules in Network”, the italic represents metabolite, the plain represents protein and the bold represents focus molecules whose expression differed by 2 fold or 0.5 fold in our experiment.

| **ID** | **Molecules in Network** | **^a^Score** | **Focus Molecules** | **Top Diseases and Functions** |
| --- | --- | --- | --- | --- |
| 1 | ***5-hydroxytryptamine***, ***acetyl-L-carnitine***, Actin, **Akr1b10**, **Aldh1a2**, Alp, ***calcifediol***, caspase, **Cdkn2aipnl**, Cg, ***cholecalciferol***, ***citric acid***, collagen, Collagen Alpha1, **Coro1a**, cytochrome C, ERK1/2, estrogen receptor, glutathione peroxidase, Hif1, ***homocitrulline***, ***indican***, **Krt20**, **Krt8**, ***kynurenic acid***, Ldh (complex), **Loxl2**, ***mannitol***, Mmp, Osteocalcin, P glycoprotein, P38 MAPK, **Rbm3**, **S100a4**, Tnf (family) | 38 | 18 | [Immunological Disease, Inflammatory Disease, Inflammatory Response] |
| 2 | ***1,4-IP2***, Akt, Ap1, **Arg1**, arginase, ***azelaic acid***, **Basp1**, Calmodulin, **Cd14**, chemokine, ***citrulline***, Creb, cytokine, F Actin, **Havcr1**, **Hp**, Ifn, Ige, IgG, IL1, IL12 (complex), IL12 (family), ***L-cysteine***, **Lcn2**, **Lcp1**, ***linoleoylethanolamide***, **Marcks**, ***N-glycolylneuraminic acid***, NMDA Receptor, **Pacs1**, ***phosphorylcholine***, Pro-inflammatory Cytokine, Tlr, **Tpm1**, **Umod** | 38 | 18 | [Immunological Disease, Inflammatory Disease, Inflammatory Response] |
| 3 | ***1-methyladenosine***, *3-hydroxyanthranilic acid*, ***5-oxo-D-proline***, *8-oxo-7-hydrodeoxyguanosine*, Acacb, acetyl-coenzyme A, Aconitase, ***adenine-riboflavin dinucleotide***, ***allantoin***, **Anxa3**, atypical protein kinase C, Bcl2l1, ***C20 acylcarnitine***, Complement, *D-glucose*, *dehydroisoandrosterone*, **Dock10**, Egfr, Elovl5, ***flavin mononucleotide***, Ggt1, **Gm12854/S100a11**, **homocitrulline**, ***L-cysteine***, ***L-cystine***, Lep, malonyl-coenzyme A, **Mup1 (includes others)**, *nicotinic acid*, Nos2, Pdia2, Plin1, Pparg, Slc13a5, ***xanthurenic acid*** | 28 | 14 | [Lipid Metabolism, Molecular Transport, Small Molecule Biochemistry] |
| 4 | ***1-22:6(4Z,7Z,10Z,13Z,16Z,19Z) lysophosphatidylcholine***, ***1-22:6(4Z,7Z,10Z,13Z,16Z,19Z) monoacylglycerol***, ***16:0/22:6(4Z,7Z,10Z,13Z,16Z,19Z) phosphatidylcholine***, ***2-22:6(4Z,7Z,10Z,13Z,16Z,19Z) lysophosphatidylethanolamine***, Aco1, adhesion molecule, App, ***butyrylcarnitine***, Casp2, Chgb, Complement, **Cp**, ***D-gluconic acid***, *dehydroisoandrosterone*, ***dihydrouracil***, Ggt1, *glutamine*, ***glycocyamine***, Hdac11, Hint1, Il1b,Il37, Il4i1, ***isethionic acid***, *kynurenine*, **Ngp**, ***nicotinamide-beta-riboside***, *nicotinic acid*, *nitrite*, Pck1, Pfkp, *phosphocreatine*, Sirt6, ***stearoylcarnitine***, Tnfrsf1a | 23 | 12 | [Amino Acid Metabolism, Lipid Metabolism, Molecular Transport] |
| 5 | ***alpha-hydroxyglutarate***, Cd3, **Cd44**, **Chil3/Chil4**, Collagen(s), Complement, Erk, Focal adhesion kinase, **Fth1**, ***guanine***, Hif, **HLA-DQA1**, Igm, Immunoglobulin, ***indican***, Insulin, Jnk, Laminin (complex), **Lgals1**, **Lrg1**, Mapk, Mek, **Myl12b**, NfkB (complex), *nicotinic acid*, PI3K (complex), PI3K (family), Pka, Pkc(s), Ras, RNA polymerase II, **Serpina3**, Src (family), Tgf beta, Vegf | 21 | 11 | [Gastrointestinal Disease, Inflammatory Disease, Inflammatory Response] |

^a^ The score is based on a p-value calculation, which calculates the likelihood that the Network Eligible Molecules that are part of a network are found therein by random chance alone. Mathematically, the score is simply the negative exponent of the right-tailed Fisher's exact test result. The score is used to rank networks according to their degree of relevance to the Focus molecules in uploaded dataset. Generally, the networks which score are more than 20 are considered reliable.
